# Tracking Control of a Maglev Vibration Isolation System Based on a High-Precision Relative Position and Attitude Model

**DOI:** 10.3390/s19153375

**Published:** 2019-08-01

**Authors:** Qianqian Wu, Bilong Liu, Ning Cui, Sifang Zhao

**Affiliations:** 1 School of Mechanical and Automotive Engineering, Qingdao University of Technology, Qingdao 266520, China; 2 College of Aerospace and Civil Engineering, Harbin Engineering University, Harbin 150001, China

**Keywords:** maglev vibration isolation, relative position and attitude, measurement model, tracking control

## Abstract

The maglev vibration isolation system exhibits excellent micro-vibration isolation performance (0.01 Hz to 100 Hz band) in the space environment. However, a collision between the base and the floating platform may occur in an ultra-low frequency range (≤0.01 Hz). To avoid collision, the relative position and attitude between the base and the floating platform needs to be accurately tracked and controlled. In this study, a novel measurement method with four groups of two-dimensional position-sensitive detectors equipped with four laser light sources was proposed. A high-precision relative position and attitude measurement model was established based on the geometric relationship of space coordinates. A proportional-differential (PD) fixed-point control algorithm was adopted to realize tracking control. The control performance of the system was evaluated through simulation. Experiments were also carried out to verify the stability of the system and the precision of the control algorithm. A maglev vibration isolation system prototype was constructed and a test system was established. The proposed relative position and attitude measurement model was verified and the six degrees of freedom relative position and attitude response of the system was tested. Based on the measurement model, the tracking control of the system was proven to have high precision.

## 1. Introduction

Scientific activities in spacecrafts have been gaining an increasing amount of attention because of the almost zero-gravity environment [1]. However, the spacecraft suffers from various disturbances, which may come from sunlight pressure, atmospheric drag, the orbit changing, and so on [2,3]. These disturbances are known as micro-vibrations, and they have a great impact on the precision of space activities. How micro-vibrations can be isolated effectively has become an important research topic.

Passive and active vibration isolation are the two main methods used to isolate micro-vibrations. The passive vibration isolation method has high reliability and good isolation performance for vibrations in middle- and high-frequency bands [4]; however, it cannot eliminate disturbance in the low-frequency range. The active vibration isolation method is used to counteract the disturbance source according to a certain control strategy [5]. The piezoelectric actuator [6,7], magnetostrictive actuators [8], and the Lorentz actuator [9] are common actuators used in an active vibration isolation system, among which the Lorentz actuator is the most promising method for creating ultra-quiet environments because of its non-contact characteristic, quick response, and wide frequency range [10,11,12,13]. Particularly when the vibration frequency is below 5 Hz, the maglev vibration isolation system, based on the Lorentz actuator, shows superior isolation performance owing to its large control stroke between the base and the floating platform [14,15,16,17]. Currently, a great deal of research is focused on the maglev vibration isolation system.

However, ultra-low frequency vibration (≤0.01 Hz) often occurs due to the randomness of environmental vibrations, which generally have a large vibration amplitude [18,19]. When the vibration amplitude is larger than the designed control stroke of the maglev vibration isolation system, a collision between the base and the floating platform occurs. Therefore, it is necessary to establish a precise mathematical model of the relative position and attitude between the base and the floating platform to realize tracking control, thereby avoiding collision.

The precision of the measurement model and the integration of the system mainly depend on the type and layout of sensors, which are the keys for tracking control. Very little research has been conducted on this problem. Visual sensors, eddy current displacement sensors, and laser displacement sensors are currently the main measuring equipment employed. A visual sensor is suitable for target tracking and motion estimation in the aerospace industry, robot vision, visual navigation [20,21,22,23], and so on. However, it will increase the complexity of the system when applied in the maglev vibration isolation system [24,25]. Eddy current displacement sensors are widely used in precision instruments due to their high sensibility and reliability [26,27]. However, they have a high requirement in terms of the shape and size of the object [28]. Laser displacement sensors also have high sensitivity and a quick response [29], whereas the volume of a laser displacement sensor is too large for a highly integrated maglev vibration isolation system. Compared with the above-mentioned sensors, the split-type position-sensitive detector (PSD) has an extremely high position resolution, which is a feasible choice to measure the relative position between the base and the platform [30,31]. Moreover, it is more suitable and economical to combine translation and rotation in the expression of coordinate system transformation without adopting additional angle sensors.

The optimization of sensor layout and the reduction of sensor quantity are guaranteed to realize a small volume and high-precision measurement. For sensors (visual sensors, eddy current displacement sensors, and laser displacement sensors) with only one-dimensional measurement data, at least six or three sensors, together with three extra angular sensors, are needed to obtain six degrees-of-freedom (DOF) measurement data [32], which is difficult to apply in the system with a small size and high integration. For two-dimensional PSD, three is enough. Limited research has been carried out on the layout of PSD. Li and Ren conducted a preliminary investigation by adopting three two-dimensional PSDs to obtain the three DOF relative positions and three DOF relative attitudes of a maglev vibration isolation system [33]. However, only the calculated model was analyzed. Experiments were not provided to verify it.

In this paper, a novel maglev vibration isolation system with four two-dimensional PSDs is designed to implement hardware redundancy and improve calculation accuracy. The six DOF relative and attitude measurement models were established, and more precise results obtained by taking advantage of redundant information. Based on the measurement model, a tracking control law of the maglev vibration isolation system was established. The tracking performance of the maglev vibration isolation at a low frequency was simulated, and experiments were also carried out to verify the measurement model and the precision of tracking control.

## 2. The Six DOF Relative Position and Attitude Measurement Model

**Figure 1:**
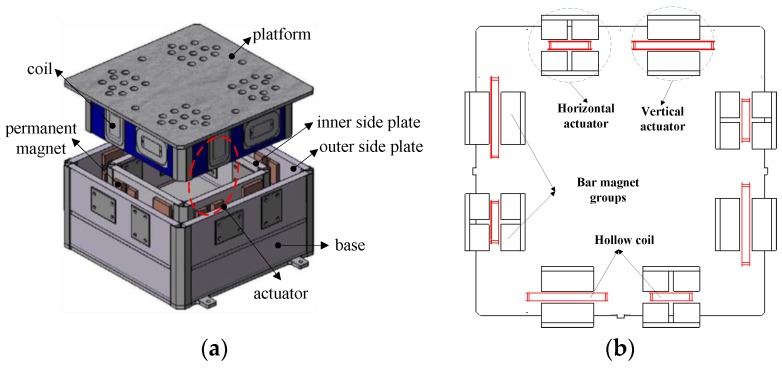
The description of six degrees-of-freedom (DOF) maglev vibration systems: (**a**) The structure of the maglev vibration isolation platform; (**b**) the layout of actuators.

A novel maglev vibration isolation platform with six DOF was designed, as shown in Figure 1. The platform is mainly composed of a double-layer base, a floating platform, and eight Lorentz actuators. Each Lorentz actuator contains a hollow coil and two groups of bar permanent magnets. The Lorentz actuator is contactless. The coils are implanted into the side plates of the floating platform, and the magnet groups are installed between the inner and outer side plates of the base. The platform is levitated between the side plates of the base. Eight actuators are installed horizontally and vertically to simultaneously generate six DOF active control forces. The relative position is at the centimeter level, and the relative attitude is at the 0.1 degree level. In order to achieve six DOF tracking controls, the measurement model with three DOF relative positions and three DOF relative attitudes should be accurately calculated.

**Figure 2:**
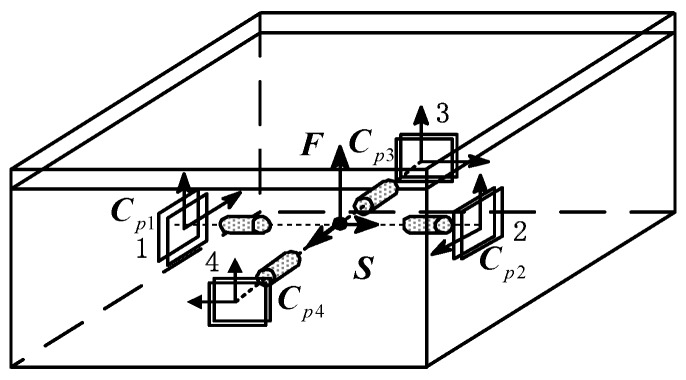
Schematic diagram of the installation position of PSDs and light sources.

The precision of the measurement model was determined by the layout and quantity of PSDs. Figure 2 shows the installation position diagram of the PSD and light sources. Four PSDs were installed on the lateral plate of the base, and four laser light sources were installed onto the lateral plate of the platform. When the base was disturbed, the position of the PSD would change accordingly. Then, the position of the light spot on the photosensitive surface of the PSD would also change. The displacement variation of the light spot on the photosensitive surface could be obtained by collecting and processing the output current of the photosensitive surface pin. According to the three-dimensional spatial geometry model, the position relationship between the displacement of the spot on the photosensitive surface and the relative motion was set up.

**Figure 3:**
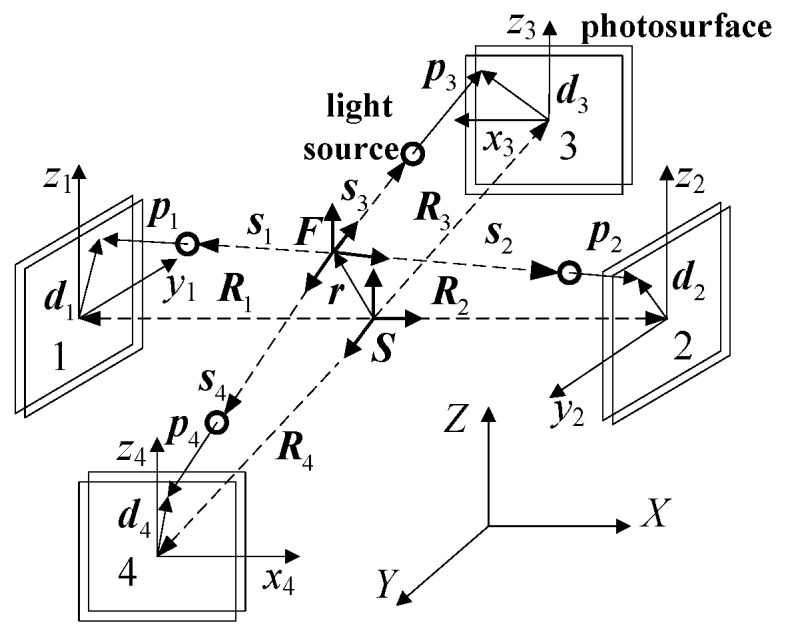
Diagram of a three-dimensional position measurement.

The diagram of the three-dimensional geometry location is shown in Figure 3. Sensors 1 and 2 were used to measure the relative position along the Z-axis and Y-axis and the relative attitude around the Z-axis and Y axis. Sensors 3 and 4 were used to measure the relative position along the Z-axis and X-axis and the relative attitude around the Z-axis and X-axis. In order to analyze the relative motion between the platform and the base, a coordinate system F was established on the platform, and the horizontal axis was parallel to the light beams from four light sources on the platform. Furthermore, a coordinate system, S, was set up on the base. The origin of the coordinate system S passes through the centroid of four PSDs and was perpendicular to the photosensitive surfaces. Moreover, coordinate systems Cp1, Cp2, Cp3, and Cp4 were established on the centroid of each PSD photosensitive surface, respectively. The Z-axis direction of these four coordinate systems was the same as that of the platform coordinate system, F.

The transformation matrix from the base coordinate system to the platform coordinate system has been defined as ***C***. The Euler attitudes are represented by $\theta_{x}$, $\theta_{y}$, and $\theta_{z}$, respectively. The transformation matrix can be derived as shown in Equation (1),

(1) $$C=\left[\begin{array}{ccc} c\theta_{y}c\theta_{z} & -c\theta_{y}s\theta_{z} & s\theta_{y}\\ s\theta_{x}s\theta_{y}c\theta_{z}+s\theta_{z}c\theta_{x} & c\theta_{x}c\theta_{z}-s\theta_{x}s\theta_{y}s\theta_{z} & -s\theta_{x}c\theta_{y}\\ s\theta_{z}s\theta_{x}-c\theta_{x}s\theta_{y}c\theta_{z} & c\theta_{x}s\theta_{y}s\theta_{z}+c\theta_{z}s\theta_{x} & c\theta_{x}c\theta_{y} \end{array}\right]$$

where, *c* = cos() and *s* = sin().

Considering the small attitude motion between the base and the platform in a maglev vibration isolation system, the transformation matrix can be simplified as:

(2) $$C=\left[\begin{array}{ccc} 1 & -\theta_{z} & \theta_{y}\\ \theta_{z} & 1 & -\theta_{x}\\ -\theta_{y} & \theta_{x} & 1 \end{array}\right]$$

The transformation matrix from the photosensitive surface of PSD to the base coordinate system has been defined as $C_{pi}$ (i = 1, 2, 3, 4). According to the installation orientation of each PSD, four transformation matrices can be written as:

(3) $$C_{p1}=\left[\begin{array}{ccc} 1 & 0 & 0\\ 0 & -1 & 0\\ 0 & 0 & 1 \end{array}\right],\;C_{p2}=\left[\begin{array}{ccc} -1 & 0 & 0\\ 0 & 1 & 0\\ 0 & 0 & 1 \end{array}\right],\;C_{p3}=\left[\begin{array}{ccc} -1 & 0 & 0\\ 0 & 1 & 0\\ 0 & 0 & 1 \end{array}\right],\;C_{p4}=\left[\begin{array}{ccc} 1 & 0 & 0\\ 0 & -1 & 0\\ 0 & 0 & 1 \end{array}\right]$$

To simplify the analysis process, the platform coordinate system is assumed to overlap with the base coordinate system at the initial state. In the base coordinate system, the relative position between the base and the platform after vibration is defined as r ($r={\left[\begin{array}{ccc} x & y & z \end{array}\right]}^{T}$), and the position vector from the origin of the base coordinate system to the photosensitive centroid of the PSD is defined as $R_{i}$ (*i* = 1, 2, 3, 4). In the platform coordinate system, the position vector from the light source to the photosensitive part of the PSD is defined as $p_{i}$ (*i* = 1, 2, 3, 4). The position vector from the origin of the platform coordinate system to the light source is defined as $s_{i}$ (*i* = 1, 2, 3, 4). In the PSD coordinate system, the position vector from the origin of the PSD to the light spot is defined as $d_{i}$ (*i* = 1, 2, 3, 4). Among those vectors, $R_{i}$ and $d_{i}$ (*i* = 1, 2, 3, 4) can be actually measured by PSD. According to the position relationship between different spatial geometric vectors, Equation (4) can be obtained and transformed in the platform coordinate system.

(4) $$p_{i}=C(C_{pi}d_{i}+R_{i}-r)-s_{i}$$

As four PSDs were used in the model, the position vectors $R_{i}$, $p_{i}$, $s_{i}$, and $d_{i}$ (*i* = 1, 2, 3, 4) can be expressed in matrix form as follows.

(5) $$R_{1,2,3,4}=\left[\begin{array}{cccc} -R_{1} & R_{2} & 0 & 0\\ 0 & 0 & -R_{3} & R_{4}\\ 0 & 0 & 0 & 0 \end{array}\right]$$

(6) $$p_{1,2,3,4}=\left[\begin{array}{cccc} -p_{1} & p_{2} & 0 & 0\\ 0 & 0 & -p_{3} & p_{4}\\ 0 & 0 & 0 & 0 \end{array}\right]$$

(7) $$s_{1,2,3,4}=\left[\begin{array}{cccc} -s_{1} & s_{2} & 0 & 0\\ 0 & 0 & -s_{3} & s_{4}\\ 0 & 0 & 0 & 0 \end{array}\right]$$

(8) $$d_{1,2,3,4}=\left[\begin{array}{cccc} 0 & 0 & d_{3x} & d_{4x}\\ d_{1y} & d_{2y} & 0 & 0\\ d_{1z} & d_{2z} & d_{3z} & d_{4z} \end{array}\right]$$

The following equations can be obtained by substituting Equations (5)–(8) into Equation (4).

(9) $$\left[\begin{array}{c} -p_{1}\\ 0\\ 0 \end{array}\right]=C\left[\begin{array}{c} -x-R_{1}\\ -d_{1y}-y\\ d_{1z}-z \end{array}\right]-\left[\begin{array}{c} -s_{1}\\ 0\\ 0 \end{array}\right]$$

(10) $$\left[\begin{array}{c} p_{2}\\ 0\\ 0 \end{array}\right]=C\left[\begin{array}{c} -x+R_{2}\\ d_{2y}-y\\ d_{2z}-z \end{array}\right]-\left[\begin{array}{c} s_{2}\\ 0\\ 0 \end{array}\right]$$

(11) $$\left[\begin{array}{c} 0\\ -p_{3}\\ 0 \end{array}\right]=C\left[\begin{array}{c} -d_{3x}-x\\ -y-R_{3}\\ d_{3z}-z \end{array}\right]-\left[\begin{array}{c} 0\\ -s_{3}\\ 0 \end{array}\right]$$

(12) $$\left[\begin{array}{c} 0\\ p_{4}\\ 0 \end{array}\right]=C\left[\begin{array}{c} d_{4x}-x\\ -y+R_{4}\\ d_{4z}-z \end{array}\right]-\left[\begin{array}{c} 0\\ s_{4}\\ 0 \end{array}\right]$$

Equation (13) can be derived by substituting Equation (2) into Equations (9)–(12).

(13) $$\{\begin{array}{c} -y-x\theta_{z}+z\theta_{x}-\theta_{x}d_{1z}-R_{1}\theta_{z}=d_{1y}\\ z-x\theta_{y}+y\theta_{x}+\theta_{x}d_{1y}-R_{1}\theta_{y}=d_{1z}\\ y+x\theta_{z}-z\theta_{x}+\theta_{x}d_{2z}-R_{2}\theta_{z}=d_{2y}\\ z-x\theta_{y}+y\theta_{x}-\theta_{x}d_{2y}+R_{2}\theta_{y}=d_{2z}\\ -x+y\theta_{z}-z\theta_{y}+\theta_{y}d_{3z}+R_{3}\theta_{z}=d_{3x}\\ z-x\theta_{y}+y\theta_{x}-\theta_{y}d_{3x}+R_{3}\theta_{x}=d_{3z}\\ x-y\theta_{z}+z\theta_{y}-\theta_{y}d_{4z}+R_{4}\theta_{z}=d_{4x}\\ z-x\theta_{y}+y\theta_{x}+\theta_{y}d_{4x}-R_{4}\theta_{x}=d_{4z} \end{array}$$

Equation (14) can be obtained by solving Equation (13).

(14) $$\left\{ \begin{array}{l} x-y\theta_{z}+z\theta_{y}=\frac{1}{2}\left[\left(d_{4x}-d_{3x}\right)+\theta_{y}\left(d_{4z}+d_{3z}\right)+\theta_{z}\left(R_{3}-R_{4}\right)\right]\\ x\theta_{z}+y-z\theta_{x}=\frac{1}{2}\left[\left(d_{2y}-d_{1y}\right)+\theta_{z}\left(R_{1}-R_{2}\right)-\theta_{x}\left(d_{2z}-d_{1z}\right)\right]\\ -x\theta_{y}+y\theta_{x}+z=\frac{1}{4}\left[\begin{array}{l} \left(d_{1z}+d_{2z}+d_{3z}+d_{4z}\right)-\theta_{y}\left(R_{2}-R_{1}-d_{3x}+d_{4x}\right)\\ -\theta_{x}\left(d_{1y}-d_{2y}-R_{4}+R_{3}\right) \end{array}\right] \end{array}\right.$$

Equation (14) can be transformed into matrix form, as

(15) $$\left[\begin{array}{ccc} 1 & -\theta_{z} & \theta_{y}\\ \theta_{z} & 1 & -\theta_{x}\\ -\theta_{y} & \theta_{x} & 1 \end{array}\right]\left[\begin{array}{c} x\\ y\\ z \end{array}\right]=\left[\begin{array}{c} m_{1}\\ m_{2}\\ m_{3} \end{array}\right]$$

where

$$\left\{ \begin{array}{l} m_{1}=\frac{1}{2}\left[\left(d_{4x}-d_{3x}\right)+\theta_{z}\left(R_{3}-R_{4}\right)+\theta_{y}\left(d_{3z}+d_{4z}\right)\right]\\ m_{2}=\frac{1}{2}\left[\left(d_{2y}-d_{1y}\right)+\theta_{z}\left(R_{2}-R_{1}\right)-\theta_{x}\left(d_{1z}+d_{2z}\right)\right]\\ m_{3}=\frac{1}{4}\left[\begin{array}{l} \left(d_{1z}+d_{2z}+d_{3z}+d_{4z}\right)-\theta_{y}\left(R_{2}-R_{1}-d_{3x}+d_{4x}\right)\\ -\theta_{x}\left(d_{1y}-d_{2y}-R_{4}+R_{3}\right) \end{array}\right] \end{array}\right.$$

Then, Equation (16) can be obtained under the condition of $\left|\begin{array}{ccc} 1 & -\theta_{z} & \theta_{y}\\ \theta_{z} & 1 & -\theta_{x}\\ -\theta_{y} & \theta_{x} & 1 \end{array}\right|\neq 0$.

(16) $$\left[\begin{array}{c} x\\ y\\ z \end{array}\right]={\left[\begin{array}{ccc} 1 & -\theta_{z} & \theta_{y}\\ \theta_{z} & 1 & -\theta_{x}\\ -\theta_{y} & \theta_{x} & 1 \end{array}\right]}^{-1}\left[\begin{array}{c} m_{1}\\ m_{2}\\ m_{3} \end{array}\right]$$

Then, the calculation formulae of the relative position between the base and the platform can be obtained as

(17) $$\{\begin{array}{c} x=m_{1}+m_{2}\theta_{z}-m_{3}\theta_{y}\\ y=m_{2}-m_{1}\theta_{z}+m_{3}\theta_{x}\\ z=m_{3}+m_{1}\theta_{y}-m_{2}\theta_{x} \end{array}$$

Similarly, by solving Equations (13), the relative attitude between the base and the platform can be obtained as

(18) $$\{\begin{array}{c} \theta_{x}=\frac{\left(d_{1z}-d_{2z}\right)\left(d_{4x}+d_{3x}\right)-\left(R_{1}+R_{2}\right)\left(d_{3z}-d_{4z}\right)}{\left(d_{4x}+d_{3x}\right)\left(d_{1y}+d_{2y}\right)-\left(R_{1}+R_{2}\right)\left(R_{4}+R_{3}\right)}\\ \theta_{y}=\frac{\left(R_{3}+R_{4}\right)\left(d_{1z}-d_{2z}\right)-\left(d_{1y}+d_{2y}\right)\left(d_{3z}-d_{4z}\right)}{\left(d_{1y}+d_{2y}\right)\left(d_{3x}+d_{4x}\right)-\left(R_{1}+R_{2}\right)\left(R_{3}+R_{4}\right)}\\ \theta_{z}=\frac{1}{2}\left[\frac{\left(d_{3x}+d_{4x}\right)-\left(d_{3z}-d_{4z}\right)\theta_{y}}{R_{3}+R_{4}}+\frac{\left(d_{2z}-d_{1z}\right)\theta_{x}-\left(d_{1y}+d_{2y}\right)}{R_{1}+R_{2}}\right] \end{array}$$

## 3. Tracking Control of the Maglev Vibration Isolation System

Tracking control of the maglev vibration isolation system was studied based on the measurement model. A state space column matrix was defined as $X\;=\;{\left[\begin{array}{cccccc} x & y & z & \theta_{x} & \theta_{y} & \theta_{z} \end{array}\right]}^{T}$, which consists of the relative position ${\left[xyz\right]}^{T}$ and the relative attitude ${\left[\theta_{x}\theta_{y}\theta_{z}\right]}^{T}$ of the system. The equations of motion for the maglev vibration isolation platform can be written as

(19) $$M_{x}\ddot{X}+C_{x}\dot{X}+K_{x}X=F_{d}+F_{u}$$

where $M_{x}$ is the mass matrix of the system, $C_{x}$ is the damping matrix, $K_{x}$ is the stiffness matrix, $F_{d}$ is the total external disturbing force matrix, and $F_{u}$ is the active control force matrix exerted on the platform.

In order to achieve motion tracking, the target value of the relative displacement $X_{0}$ between the base and the platform should be a constant ***C***_0_, associated with system parameters. The target value of the relative velocity ${\dot{X}}_{0}$ and the relative acceleration ${\ddot{X}}_{0}$ should be **0**.

Defining $e_{p}$ as the relative displacement error, it can then be expressed as

(20a) $$e_{p}=X_{0}-X$$

Therefore, the relative velocity error and the relative acceleration error can be written as

(20b) $${\dot{e}}_{p}={\dot{X}}_{0}-\dot{X}$$

(20c) $${\ddot{e}}_{p}={\ddot{X}}_{0}-\ddot{X}$$

Equation (20a–c) can be transformed as

(21a) $$X=C_{0}-e_{p}$$

(21b) $$\dot{X}=0-{\dot{e}}_{p}$$

(21c) $$\ddot{X}=0-{\ddot{e}}_{p}$$

Substituting Equation (21a–c) into Equation (19), the equation of motion of the maglev vibration isolation system can be transformed as

(22) $$M_{x}{\ddot{e}}_{p}+C_{x}{\dot{e}}_{p}+K_{x}e_{p}+F_{u}=-F_{d}+K_{x}C_{0}$$

The active control force based on a PD-fixed control algorithm is defined as

(23) $$F_{u}=K_{d}{\dot{e}}_{p}{+K}_{p}e_{p}-F_{d}+K_{x}C_{0}$$

where $K_{d}$ is the differential coefficient and $K_{p}$ is the proportionality coefficient.

Then, Equation (22) can be reduced to

(24) $$M_{x}{\ddot{e}}_{p}+\left(C_{x}{+K}_{d}\right){\dot{e}}_{p}+\left(K_{x}{+K}_{p}\right)e_{p}=0$$

A Lyapunov function is defined as

(25) $$V=\frac{1}{2}{\dot{e}}_{p}{}^{T}M_{x}{\dot{e}}_{p}+\frac{1}{2}e_{p}{}^{T}K_{p}e_{p}$$

The matrix $M_{x}$ and $K_{p}$ are positive definites, and ***V*** is globally positive. Then, the differential of ***V*** yields

(26) $${\dot{V}=\dot{e}}_{p}{}^{T}M_{x}{\ddot{e}}_{p}+\frac{1}{2}{\dot{e}}_{p}{}^{T}{\dot{M}}_{x}{\dot{e}}_{p}+{\dot{e}}_{p}{}^{T}K_{p1}e_{p}$$

Based on the small attitude hypothesis and Equation (22), Equation (24) can be written as

(27) $${\dot{V}=\dot{e}}_{p}{}^{T}\left(M_{x}{\ddot{e}}_{p}+K_{p}e_{p}\right)=-{\dot{e}}_{p}{}^{T}\left(C_{x}+K_{d}\right){\dot{e}}_{p}$$

In order to decrease the control quantities exponentially, the damping ratio of the system is required to be greater than or equal to 1. Therefore, it can be obtained that $C_{x}+K_{d}\geq 2M_{x}\sqrt{M_{x}{}^{-1}K_{p}}$, indicating that $C_{x}+K_{d}$ must be a positive definite. According to Equation (27), there is $\dot{V}\leq 0$.

Based on the above analysis, when $\dot{V}\equiv 0$, there must be ${\dot{e}}_{p}\equiv 0$. Thereby, ${\ddot{e}}_{p}\equiv 0$.

Taking ${\dot{e}}_{p}\equiv 0$ and ${\ddot{e}}_{p}\equiv 0$ into Equation (22), it can be simplified as $\left(K_{x}{+K}_{p}\right)e_{p}=0$. As $K_{x}$ and $K_{p}$ are positive definites, then $e_{p}\equiv 0$. According to LaSalle’s theorem, $\left(e_{p},{\dot{e}}_{p}\right)\equiv \left(\begin{array}{cc} 0, & 0 \end{array}\right)$ is the globally asymptotically stable equilibrium point, which enables any inertial condition to reach the target [34].

**Figure 4:**
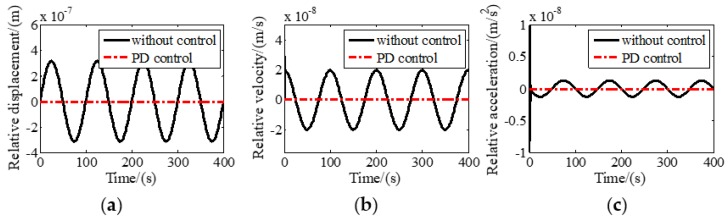
Relative motion with and without tracking control along *Z* direction: (**a**) Relative displacement; (**b**) relative linear velocity; (**c**) relative linear acceleration.

**Figure 5:**
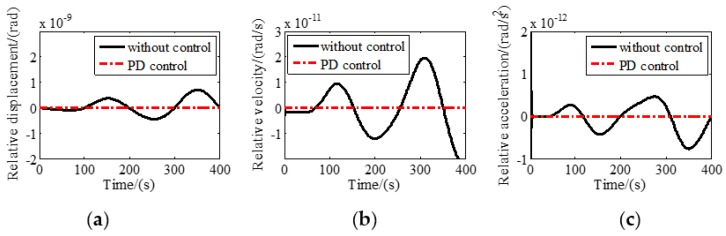
Relative motion with and without tracking control around *Z* direction: (**a**) Relative displacement; (**b**) relative velocity; (**c**) relative acceleration.

A control program was constructed in MATLAB/SIMULINK to simulate the tracking performance of the six DOF maglev vibration isolation system. Sinusoidal disturbance with a frequency of 0.01 Hz and amplitude of 3 mm was assumed to be exerted on the base along the Z axis. Relative motions with and without tracking control along and around Z direction are shown in Figure 4 and Figure 5, respectively. It can be seen that the relative displacement, relative velocity, and relative acceleration between the base and the platform approach zero quickly in a very short period of time, indicating that the maglev vibration isolation system has good tracking performance at 0.01 Hz.

## 4. Experiments

**Figure 6:**
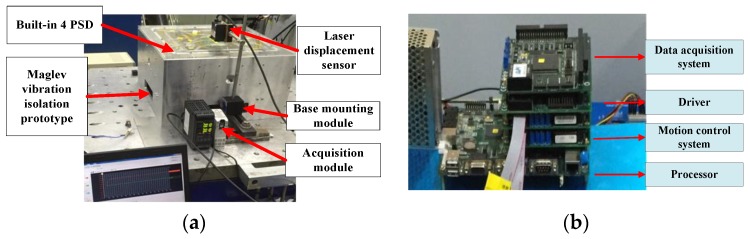
Experimental set-up: (**a**) Prototype and test component; (**b**) hardware of the maglev vibration isolation system.

**Figure 7:**
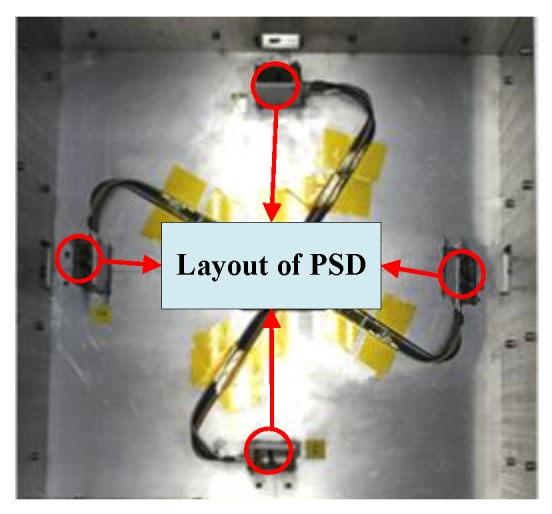
Layout of four PSDs.

Experiments were conducted to evaluate the validity of the above model. A prototype of the maglev vibration isolation platform was manufactured. The mass and moment of inertia of the manufactured floating platform are listed in Table 1. In addition, the identified force constants, the equivalent stiffness matrix, and the damping matrix were identified as shown in Table 2 and Table 3, respectively. The Lorentz forces can be obtained by multiplying force constants with currents. The experimental device is shown in Figure 6. The base of the maglev vibration isolation prototype was fixed by the installation module. A laser displacement sensor (KEYENCE LK-H025) equipped with an acquisition module was used to measure the displacement of the platform. The light spot of the laser displacement sensor was focused on the center of the platform. Four two-dimensional PSDs (DL400-7-PCBA) were installed onto the base, and four light sources (LDM635-5LT) were fixed onto the floating platform. The layout of the PSDs is shown in Figure 7.

### 4.1. Verification of the Relative Position Measurement Model

**Figure 8:**
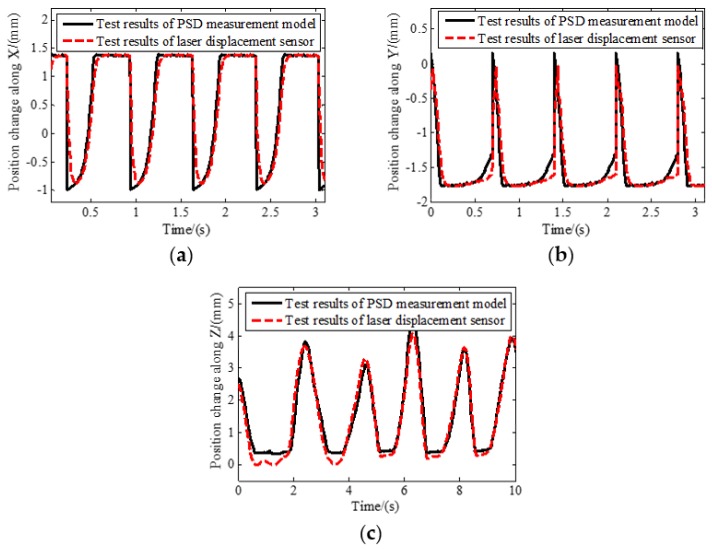
Comparison of relative position changes: (**a**) Position change along *X* direction; (**b**) position change along *Y* direction; (**c**) position change along *Z* direction.

The floating platform was pushed back and forth along the *X* axis, *Y* axis, and *Z* axis, respectively. During the movement, the displacement of the spot on the photosensitive surface was measured by PSDs. The motion information between the base and the platform was calculated by the relative position measurement model. To verify the proposed measurement model, a laser displacement sensor was used to measure the displacement of the floating platform. A comparison of the test results of the PSD and laser displacement sensor is shown in Figure 8.

As the reciprocating movement process of the floating platform is relatively random and the installation precision requirement of laser displacement sensor is not high, measuring errors exist. The maximum error between the calculated results based on PSDs and the laser displacement sensor is shown in Table 4. In general, the position change trend and the amplitude of the results of two kinds of sensors are consistent, proving that the relative position measurement model is accurate.

### 4.2. Relative Position and Attitude Dynamic Response Test

**Figure 9:**
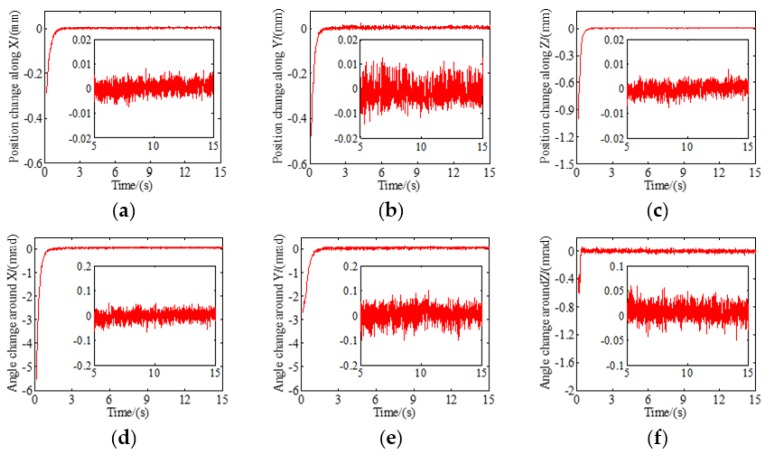
Test of relative position and attitude with six DOFs: (**a**) Position change along *X*; (**b**) position change along *Y*; (**c**) position change along *Z*; (**d**) attitude change around *X*; (**e**) attitude change around *Y*; (**f**) attitude change around *Z*.

According to the proposed tracking control law of the maglev vibration isolation system, the dynamic response of relative position and attitude can be obtained. The physical zero of the photosensitive surface of the PSD was set as the target zero. Then, the PD parameters of the control law were adjusted by trying from small to large. The proportionality coefficient of each DOF was set as 60 and the differential coefficient of each DOF was set as 180. The floating platform could move from the initial position to the target position under the control of eight actuators. According to the proposed measurement model, the response results of the relative position and attitude could be calculated. It can be seen in Figure 9 that the relative position and attitude of the maglev vibration isolation system can reach the target position within 1 to 2 s, and the overshoot is small. The steady-state response errors along and around the *X* axis, *Y* axis, and *Z* axis are 13.85 μm, 16.27 μm, 10.55 μm, 108.3 μrad, 143.21 μrad, and 103.4 μrad, respectively. The results indicate that the system can quickly reach the target under tracking control based on the measurement model.

**Figure 10:**
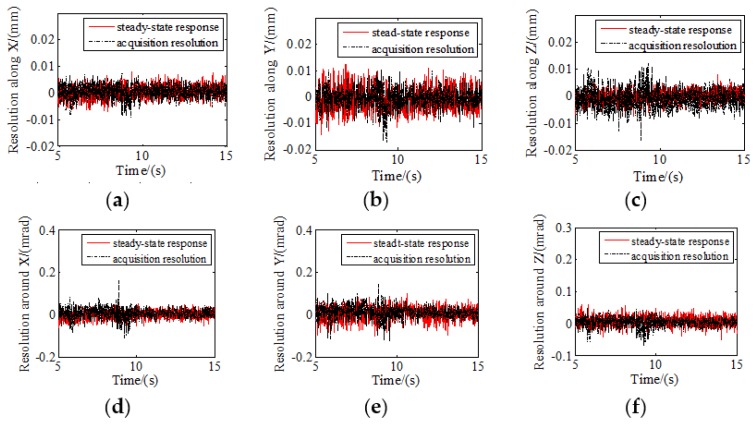
Resolution comparison of relative position and attitude with six DOFs: (**a**) Resolution along *X*; (**b**) resolution along *Y*; (**c**) resolution along *Z*; (**d**) resolution around *X*; (**e**) resolution around *Y*; (**f**) resolution around *Z*.

The acquisition resolution of the PSDs was tested in a static state, and the data was compared with the steady-state response results from 5 to 15 s (Figure 10). The control resolution of the six DOFs is consistent with the acquisition resolution in the static state, which confirmed that the tracking control has high precision.

The root mean square errors between the steady-state response and acquisition resolution are listed in Table 5, and the maximum root mean square error is 0.0187. The tracking control is proven to have high precision as the control resolution of six DOFs is consistent with the acquisition resolution in the static state.

## 5. Conclusions

In this paper, a novel measurement method with four groups of two-dimensional PSDs equipped with four laser light sources has been proposed. According to the geometric relationship of space coordinates, three DOF relative positions and three DOF attitude measurement models of maglev vibration isolation systems were developed. A tracking control model of the maglev vibration isolation system based on the measurement model was also established. A control program was developed, and the tracking performance was simulated when the disturbing frequency was 0.01 Hz. The simulation results showed that the system had excellent tracking performance. A prototype of the maglev vibration isolation system was manufactured and experiments were conducted to verify the proposed measurement model. By comparing the test results of the laser displacement sensor and PSDs, it could be concluded that the relative position and attitude measurement model is accurate. The dynamic response of the maglev vibration isolation system shows that, based on the proposed measurement model, the tracking control has high precision.

## Figures and Tables

**Table 1:** Mass and moment of inertia of the floating platform.

Description	Value
*m*/Kg	5.42
*J_xx_*/Kg·m^2^	0.1005
*J_xy_*/Kg·m^2^	−4.776 × 10^−6^
*J_xz_*/Kg·m^2^	0
*J_yy_*/Kg·m^2^	0.1005
*J_yz_*/Kg·m^2^	0
*J_zz_/*Kg·m^2^	0.195

**Table 2:** Force constant of actuators.

Description	Value (N/A)
Actuator 1	8.0911
Actuator 2	8.0911
Actuator 3	8.2748
Actuator 4	8.2748
Actuator 5	8.0911
Actuator 6	8.0911
Actuator 7	7.9093
Actuator 8	7.9093

**Table 3:** Equivalent stiffness matrix and damping matrix of cables.

Description	Stiffness Matrix (N/mm)	Damping Matrix (N·s/mm)
Power cable	[−22.16 0 45.73;0 7.44 0; −45.25 0 61.85]	[0.0013 0 −0.0011;0 −0.0106 0;0.0102 0 0.0305]
Signal cable	[−30.24 0 62.35;0 8.78 0; −62.67 0 91.62]	[−0.025 0 0.0016;0 −0.0023 0; −0.012 0 −0.0253]

**Table 4:** Maximum error between calculated results based on PSDs and laser displacement sensor.

Along X/mm	Along Y/mm	Along Z/mm
0.109	0.129	0.585

**Table 5:** Comparison of the root mean square errors between steady-state response and acquisition resolution.

Along X	Along Y	Along Z	Around X	Around Y	Around Z
0.0078	0.0108	0.0187	1.03 × 10^−4^	7.87 × 10^−5^	2.47 × 10^−5^
